# Crystal structure and Hirshfeld surface analysis of 4-amino­pyridinium thio­cyanate–4-amino­pyridine (1/1)

**DOI:** 10.1107/S2056989020011445

**Published:** 2020-08-28

**Authors:** M. Renugadevi, A. Sinthiya, Kumaradhas Poomani, Suganya Suresh

**Affiliations:** a Department of Physics, St Josephs College (Autonomous), Affiliated to Bharathidasan University, Tiruchirappalli 620002, Tamil Nadu, India; b Laboratory of Biocrystallography and Computational Molecular Biology, Department of Physics, Periyar University, Salem 636 011, Tamil Nadu, India

**Keywords:** crystal structure, 4-amino­pyridinium, thio­cyanate, 4-amino­pyridine, hydrogen bonding

## Abstract

In the crystals of the title compound, C_5_H_7_N_2_^+^·CNS^−^·C_5_H_6_N_2_, the components are linked by three N—H⋯N and two N—H⋯S hydrogen bonds, resulting in two inter­penetrating three-dimensional networks. Hirshfeld surface analysis shows that the most important contributions to the crystal packing are from H⋯H (36.6%), C⋯H/H⋯C (20.4%), S⋯H/H⋯S (19.7%) and N⋯H/H⋯N (13.4%) inter­actions.

## Abstract 2

In the crystal of the title compound, the 4-amino­pyridine mol­ecules, 4-amino­pyridinium and thio­cyanate ions are held together by N—H⋯S and N—H⋯N hydrogen bonds.

## Chemical context   

Processes based on metathesis reactions are a greener alternative for the synthesis of organic materials, avoiding haza­rdous pollution to the environment (Grubbs, 2003 ▸). A Nobel prize was awarded for the development of metathesis reactions used for the synthesis of organic mol­ecules. Later, new pharmaceuticals and agrochemical materials were developed using this reaction.
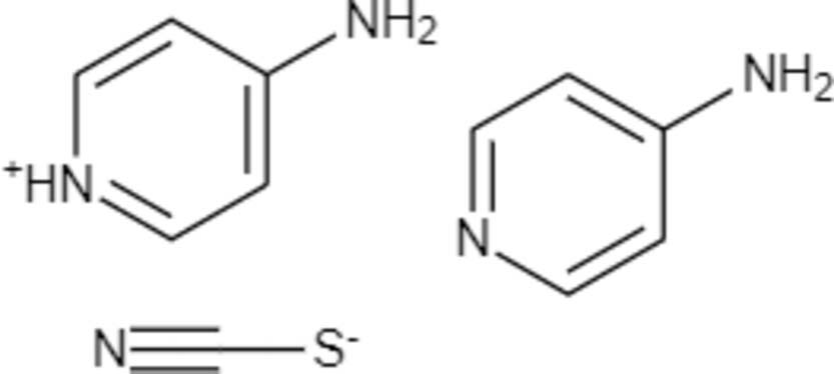


In order to access important sulfur-containing compounds, organic thio­cyanates play vital role as synthetic inter­mediates (Castanheiro *et al.*, 2016 ▸). The versatile thio­cyanate ion can join to the reaction centre of a suitable cation or neutral mol­ecule through the S or N atom, resulting in the assembly of supra­molecular compounds (Lee *et al.*, 2017 ▸). For example, the crystal of 2-amino­cyclo­hexan-1-aminium thio­cyanate involves N—H⋯S and N—H⋯N inter­actions between the thio­cyanate anion and the amine and aminium groups, leading to the formation of a two-dimensional network (Salem *et al.*, 2012 ▸). 4-Amino­pyridine has many biological applications, especially in treating neurological problems. For example, it acts as a potassium channel blocker (Schwid *et al.*, 1997 ▸). With this background, the present work is carried out and the results are reported here.

## Structural commentary   

**Figure 1:**
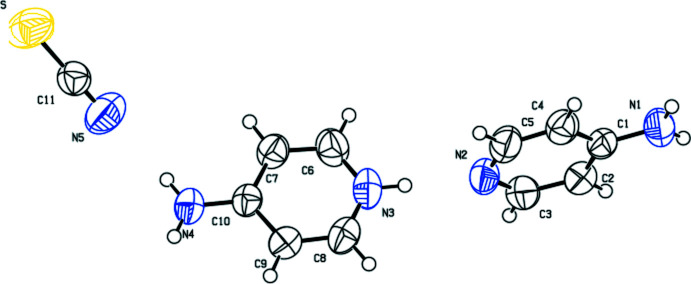
View of the asymmetric unit of the title compound.

The asymmetric unit of the title compound is composed of one 4-amino­pyridine mol­ecule, one 4-amino­pyridinium cation and one thio­cyanate anion as shown in Fig. 1 ▸. The cation forms hydrogen bonds with the neutral mol­ecule and with the anion (Table 1 ▸). The bond lengths and angles in neutral 4-amino­pyridine are similar to those in a previous report (Anderson *et al.*, 2005 ▸), but the bond angle at the pyridine N3 atom is increased to 119.47 (14)° due to the hydrogen-bonding inter­action. The thio­cyanate anion is linear with an N5—C11—S bond of 177.85 (18)°. All bond lengths and angles in the amino­pyridinium cation are within the normal ranges (Fun *et al.*, 2010 ▸).

## Supra­molecular features   

**Figure 2:**
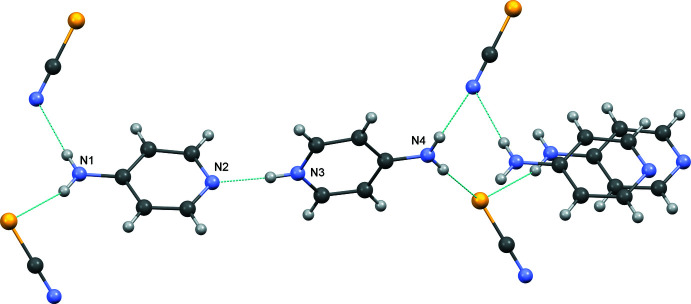
Hydrogen bonds in the crystal of the title compound.

**Figure 3:**
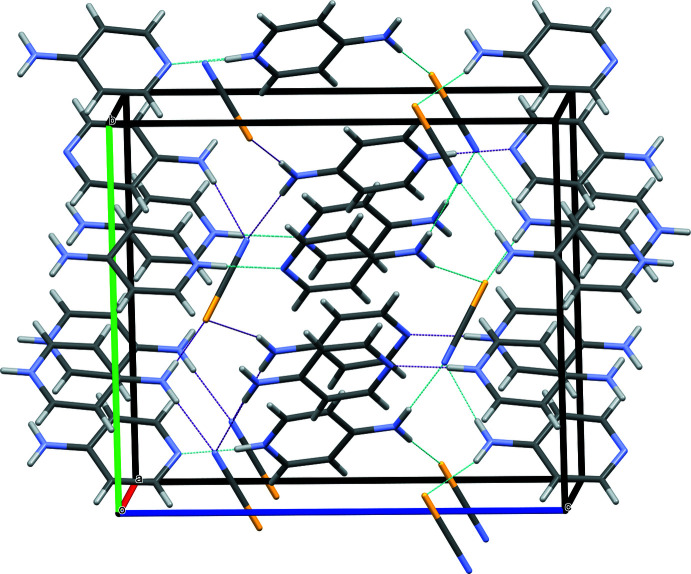
Crystal packing diagram of the title compound showing two inter­penetrating 3D nets of hydrogen bonds presented as blue and purple dotted lines.

In the crystal, the 4-amino­pyridinium cation and 4-amino­pyridine mol­ecule are linked by a strong N—H⋯N hydrogen bond (Table 1 ▸). The thio­cyanate ions act as bridges, each of them forming two N⋯H—N and two S⋯H—N hydrogen bonds (Fig. 2 ▸). As a result, two inter­penetrating three-dimensional nets of hydrogen bonds are formed, as shown in Fig. 3 ▸. The short inter­planar distance of 3.3419 (7) Å between the mean planes of two 4-amino­pyridine mol­ecules related by an inversion center indicates a π–π inter­action [*Cg*⋯*Cg*(1 − *x*, −*y*, −*z*) = 3.7635 (13) Å where *Cg* is the centroid of the N2/C1–C5 ring.

## Hirshfeld surface analysis   

**Figure 4:**
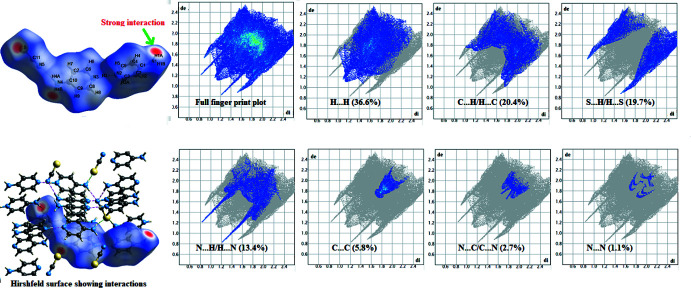
Hirshfeld surface plotted over *d*_norm_ and two-dimensional fingerprint plots for the title compound.

To qu­antify the inter­molecular contacts in the title structure, the Hirshfeld surface and two-dimensional fingerprint plots were calculated using *Crystal Explorer* (Turner *et al.*, 2017 ▸). The Hirshfeld surface mapped over *d*_norm_ is depicted in Fig. 4 ▸, where the red regions make apparent hydrogen bonds in this structure. The intensity of the red color is higher for N1—H1*A*⋯S, indicating the strongest inter­action as compared to other red spots on the Hirshfeld surface. The fingerprint plots show that the largest contributions are from H⋯H (36.6%), C⋯H/H⋯C (20.4%), S⋯H/H⋯S (19.7%) and N⋯H/H⋯N (13.4%) inter­actions. Other inter­actions contributing to the crystal packing are C⋯C (5.8%), N⋯C/C⋯N (2.7%), N⋯N (1.1%), N⋯S/S⋯N (0.2%) and S⋯C/C⋯S (0.2%).

## Database survey   

A search of the Cambridge Crystallographic Database (CSD, version 5.40, update of September 19; Groom *et al.*, 2016 ▸) was undertaken for structures containing 4-amino­pyridine and for thio­cyanate ions in the salts with organic ammonium cations. The room-temperature structure of 4-amino­pyridine was reported by Chao & Schempp (1977 ▸). Anderson *et al.* (2005 ▸) redetermined the structure at 150 K and reported that pyramidalization occurs at the amino N atom, with the N atom displaced from the plane of the three C/H/H atoms to which it is bonded. An N—H⋯N(pyridine) inter­action links the mol­ecules in a head-to-tail manner, forming zigzag chains along the *c*-axis direction. This is in contrast to the structure of the title compound, where N—H⋯N(pyridine) inter­actions link the mol­ecules in a tail-to-tail manner. van Rooyan & Boeyens (1975 ▸) reported the SCH ions in sodium thio­cyanate to be linear within experimental error. reported that in 2-amino­cyclo­hexan-1-aminium thio­cyanate (Salem *et al.*, 2012 ▸), the thio­cyanate anion is involved in N—H⋯S and N—H⋯N inter­actions with both the amine and the aminium N atoms. Bagabas *et al.* (2015 ▸) reported that cyclo­hexyl ammonium thio­cyanate has slightly a distorted chair conformation and that the mol­ecules are linked by N—H⋯N and N—H⋯S hydrogen-bonding inter­actions. In bis­[(18-crown-6-κ^6^ *O*)sodium] (18-crown-6-1κ^6^ *O*)-μ-thio­cyanato-1:2κ^2^ *S*:*N*-penta­thio­­cyanato-2κ^5^ *N*-indate(III)sodium 1,2-di­chloro­ethane ses­qui­solvate (Kong, 2009 ▸), the metal atom is in a six-coordinated octa­hedral environment, bounded to the N atoms of six thio­cyanate ions and the crystal packing exhibits no significant short inter­molecular contacts. In the title compound the N—H⋯N and N—H⋯S hydrogen bonds link the mol­ecules into centrosymmetric structure and 4-amino­pyridine is connected to the SCN ion by N—H⋯N hydrogen bonds.

## Synthesis and crystallization   

4-Amino­pyridine and sodium thio­cyanate were purchased from Merck. A solution of equimolar amounts of 4-amino­pyridine and sodium thio­cyanate in double-distilled water was stirred intensively for nearly 4 h, filtered with Whatman filter paper and allowed to evaporate at room temperature. Colourless needle-like crystals of the title compound were obtained after a period of seven days.

## Refinement   

Crystal data, data collection and structure refinement details are summarized in Table 2 ▸. All H atoms were placed in idealized positions (C—H = 0.93 Å, N—H = 0.86 Å) and treated as riding with *U*_iso_(H) = 1.2*U*_eq_(C,N).

## Supplementary Material

Crystal structure: contains datablock(s) global, I. DOI: 10.1107/S2056989020011445/yk2137sup1.cif

Structure factors: contains datablock(s) I. DOI: 10.1107/S2056989020011445/yk2137Isup2.hkl

Supporting information file. DOI: 10.1107/S2056989020011445/yk2137Isup4.mol

Supporting information file. DOI: 10.1107/S2056989020011445/yk2137Isup4.cml

CCDC reference: 2024317

Additional supporting information:  crystallographic information; 3D view; checkCIF report

## Figures and Tables

**Table 1:** Hydrogen-bond geometry (Å, °)

*D*—H⋯*A*	*D*—H	H⋯*A*	*D*⋯*A*	*D*—H⋯*A*
N4—H4*A*⋯S^i^	0.86	2.58	3.4222 (17)	165
N4—H4*B*⋯N5	0.86	2.10	2.952 (2)	168
N3—H3⋯N2	0.86	1.83	2.688 (2)	172
N1—H1*A*⋯S^ii^	0.86	2.62	3.4498 (18)	162
N1—H1*B*⋯N5^iii^	0.86	2.23	3.083 (3)	172

Symmetry codes: (i) 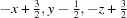; (ii) 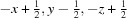; (iii) 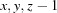.

**Table 2:** Experimental details

Crystal data	
Chemical formula	C_5_H_7_N_2_^+^·CNS^−^·C_5_H_6_N_2_
*M*_r_	247.32
Crystal system, space group	Monoclinic, *P*2_1_/*n*
Temperature (K)	293
*a*, *b*, *c* (Å)	7.9047 (19), 12.138 (2), 13.959 (3)
β (°)	94.670 (8)
*V* (Å^3^)	1334.9 (5)
*Z*	4
Radiation type	Mo *K*α
μ (mm^−1^)	0.23
Crystal size (mm)	0.70 × 0.44 × 0.34

Data collection	
Diffractometer	Bruker APEXII CCD
Absorption correction	Multi-scan (*SADABS*; Sheldrick, 2014 ▸)
*T*_min_, *T*_max_	0.86, 0.93
No. of measured, independent and observed [*I* > 2σ(*I*)] reflections	15272, 3295, 2604
*R*_int_	0.023
(sin θ/λ)_max_ (Å^−1^)	0.667

Refinement	
*R*[*F* ^2^ > 2σ(*F* ^2^)], *wR*(*F* ^2^), *S*	0.053, 0.148, 1.03
No. of reflections	3295
No. of parameters	154
H-atom treatment	H-atom parameters constrained
Δρ_max_, Δρ_min_ (e Å^−3^)	0.19, −0.27

Computer programs: *APEX2* (Bruker, 2005 ▸), *SAINT* (Bruker, 2001 ▸), *SHELXT2018/2* (Sheldrick, 2015*a* ▸), *SHELXL2018/3* (Sheldrick, 2015*b* ▸) and *ORTEP-3 for Windows* and *WinGX* publication routines (Farrugia, 2012 ▸).

## supplementary crystallographic information

### Crystal data

**Table:**

C_5_H_7_N_2_^+^·CNS^−^·C_5_H_6_N_2_	*F*(000) = 520
*M**_r_* = 247.32	*D*_x_ = 1.231 Mg m^−^^3^
Monoclinic, *P*2_1_/*n*	Mo *K*α radiation, λ = 0.71075 Å
*a* = 7.9047 (19) Å	Cell parameters from 15272 reflections
*b* = 12.138 (2) Å	θ = 3.0–28.0°
*c* = 13.959 (3) Å	µ = 0.23 mm^−^^1^
β = 94.670 (8)°	*T* = 293 K
*V* = 1334.9 (5) Å^3^	Needle, colourless
*Z* = 4	0.70 × 0.44 × 0.34 mm

### Data collection

**Table:**

Bruker APEXII CCD diffractometer	2604 reflections with *I* > 2σ(*I*)
Radiation source: fine-focus sealed tube	*R*_int_ = 0.023
φ and ω scans	θ_max_ = 28.3°, θ_min_ = 2.9°
Absorption correction: multi-scan (SADABS; Sheldrick, 2014)	*h* = −10→10
*T*_min_ = 0.86, *T*_max_ = 0.93	*k* = −16→14
15272 measured reflections	*l* = −18→18
3295 independent reflections	

### Refinement

**Table:**

Refinement on *F*^2^	Primary atom site location: structure-invariant direct methods
Least-squares matrix: full	Secondary atom site location: difference Fourier map
*R*[*F*^2^ > 2σ(*F*^2^)] = 0.053	Hydrogen site location: inferred from neighbouring sites
*wR*(*F*^2^) = 0.148	H-atom parameters constrained
*S* = 1.03	*w* = 1/[σ^2^(*F*_o_^2^) + (0.0676*P*)^2^ + 0.2492*P*] where *P* = (*F*_o_^2^ + 2*F*_c_^2^)/3
3295 reflections	(Δ/σ)_max_ < 0.001
154 parameters	Δρ_max_ = 0.19 e Å^−^^3^
0 restraints	Δρ_min_ = −0.27 e Å^−^^3^

### Special details

**Table:**

Geometry. All esds (except the esd in the dihedral angle between two l.s. planes) are estimated using the full covariance matrix. The cell esds are taken into account individually in the estimation of esds in distances, angles and torsion angles; correlations between esds in cell parameters are only used when they are defined by crystal symmetry. An approximate (isotropic) treatment of cell esds is used for estimating esds involving l.s. planes.

### Fractional atomic coordinates and isotropic or equivalent isotropic displacement parameters (Å^2^)

**Table:**

	*x*	*y*	*z*	*U*_iso_*/*U*_eq_	
S	0.53196 (8)	0.54205 (5)	0.81261 (6)	0.1064 (3)	
N4	0.60108 (18)	0.16957 (12)	0.60040 (9)	0.0665 (4)	
H4A	0.680133	0.129475	0.628322	0.080*	
H4B	0.548015	0.216341	0.633239	0.080*	
N3	0.47226 (19)	0.13478 (13)	0.31204 (9)	0.0692 (4)	
H3	0.444115	0.126754	0.251630	0.083*	
N2	0.4113 (2)	0.11863 (13)	0.12028 (10)	0.0723 (4)	
N1	0.3084 (2)	0.15215 (13)	−0.17439 (10)	0.0761 (4)	
H1A	0.234378	0.110319	−0.204780	0.091*	
H1B	0.360945	0.201212	−0.205073	0.091*	
C10	0.56072 (18)	0.15921 (12)	0.50609 (10)	0.0513 (3)	
C1	0.34225 (19)	0.14074 (13)	−0.07807 (10)	0.0568 (4)	
C7	0.4312 (2)	0.22270 (14)	0.45860 (11)	0.0620 (4)	
H7	0.373318	0.274750	0.492303	0.074*	
C9	0.6444 (2)	0.08397 (13)	0.45041 (11)	0.0599 (4)	
H9	0.731830	0.040635	0.478561	0.072*	
N5	0.4673 (3)	0.34464 (16)	0.72012 (13)	0.0982 (6)	
C8	0.5971 (2)	0.07464 (15)	0.35512 (12)	0.0649 (4)	
H8	0.653756	0.024592	0.318724	0.078*	
C2	0.2604 (2)	0.06267 (13)	−0.02580 (12)	0.0650 (4)	
H2	0.180504	0.015765	−0.056717	0.078*	
C11	0.4971 (2)	0.42600 (16)	0.75820 (12)	0.0656 (4)	
C4	0.4626 (2)	0.20694 (14)	−0.02711 (12)	0.0638 (4)	
H4	0.521971	0.260152	−0.058647	0.077*	
C3	0.2976 (2)	0.05505 (15)	0.07087 (12)	0.0713 (5)	
H3A	0.240361	0.002503	0.104414	0.086*	
C6	0.3912 (2)	0.20756 (16)	0.36331 (13)	0.0718 (5)	
H6	0.304243	0.249325	0.332609	0.086*	
C5	0.4917 (2)	0.19239 (16)	0.06949 (13)	0.0712 (5)	
H5	0.573045	0.236775	0.102281	0.085*	

### Atomic displacement parameters (Å^2^)

**Table:**

	*U*^11^	*U*^22^	*U*^33^	*U*^12^	*U*^13^	*U*^23^
S	0.0858 (4)	0.0832 (4)	0.1442 (6)	−0.0020 (3)	−0.0260 (4)	−0.0304 (3)
N4	0.0740 (9)	0.0703 (8)	0.0540 (7)	0.0003 (7)	−0.0030 (6)	−0.0093 (6)
N3	0.0759 (9)	0.0846 (10)	0.0466 (7)	−0.0138 (8)	0.0026 (6)	−0.0061 (6)
N2	0.0837 (10)	0.0825 (10)	0.0502 (7)	0.0200 (8)	0.0031 (7)	−0.0076 (7)
N1	0.0919 (10)	0.0808 (10)	0.0546 (8)	−0.0061 (8)	−0.0003 (7)	−0.0010 (7)
C10	0.0518 (7)	0.0510 (7)	0.0513 (7)	−0.0127 (6)	0.0052 (6)	−0.0041 (6)
C1	0.0602 (8)	0.0580 (8)	0.0524 (8)	0.0128 (6)	0.0052 (6)	−0.0065 (6)
C7	0.0575 (8)	0.0665 (9)	0.0620 (9)	0.0026 (7)	0.0052 (7)	−0.0096 (7)
C9	0.0607 (8)	0.0562 (8)	0.0629 (9)	−0.0008 (7)	0.0065 (7)	−0.0045 (7)
N5	0.1254 (16)	0.0796 (12)	0.0945 (13)	−0.0078 (10)	0.0381 (11)	−0.0197 (10)
C8	0.0708 (10)	0.0654 (9)	0.0606 (9)	−0.0094 (8)	0.0180 (7)	−0.0126 (7)
C2	0.0735 (10)	0.0593 (9)	0.0618 (9)	0.0019 (7)	0.0027 (7)	−0.0071 (7)
C11	0.0665 (9)	0.0691 (10)	0.0618 (9)	0.0046 (8)	0.0096 (7)	0.0052 (8)
C4	0.0604 (9)	0.0683 (9)	0.0630 (9)	0.0036 (7)	0.0075 (7)	−0.0066 (7)
C3	0.0853 (12)	0.0663 (10)	0.0635 (10)	0.0089 (9)	0.0133 (9)	0.0021 (8)
C6	0.0666 (10)	0.0843 (12)	0.0631 (10)	0.0041 (9)	−0.0034 (7)	0.0008 (9)
C5	0.0679 (10)	0.0794 (11)	0.0648 (10)	0.0082 (9)	−0.0028 (8)	−0.0180 (9)

### Geometric parameters (Å, º)

**Table:**

S—C11	1.613 (2)	C1—C4	1.395 (2)
N4—C10	1.3351 (19)	C7—C6	1.354 (2)
N4—H4A	0.8600	C7—H7	0.9300
N4—H4B	0.8600	C9—C8	1.357 (2)
N3—C6	1.333 (2)	C9—H9	0.9300
N3—C8	1.331 (2)	N5—C11	1.137 (2)
N3—H3	0.8600	C8—H8	0.9300
N2—C3	1.334 (2)	C2—C3	1.361 (2)
N2—C5	1.335 (2)	C2—H2	0.9300
N1—C1	1.3565 (19)	C4—C5	1.361 (2)
N1—H1A	0.8600	C4—H4	0.9300
N1—H1B	0.8600	C3—H3A	0.9300
C10—C9	1.400 (2)	C6—H6	0.9300
C10—C7	1.404 (2)	C5—H5	0.9300
C1—C2	1.388 (2)		
			
C10—N4—H4A	120.0	C10—C9—H9	120.2
C10—N4—H4B	120.0	N3—C8—C9	122.14 (15)
H4A—N4—H4B	120.0	N3—C8—H8	118.9
C6—N3—C8	119.47 (14)	C9—C8—H8	118.9
C6—N3—H3	120.3	C3—C2—C1	119.71 (16)
C8—N3—H3	120.3	C3—C2—H2	120.1
C3—N2—C5	116.33 (15)	C1—C2—H2	120.1
C1—N1—H1A	120.0	N5—C11—S	177.85 (18)
C1—N1—H1B	120.0	C5—C4—C1	118.94 (17)
H1A—N1—H1B	120.0	C5—C4—H4	120.5
N4—C10—C9	121.56 (14)	C1—C4—H4	120.5
N4—C10—C7	121.36 (14)	N2—C3—C2	123.74 (17)
C9—C10—C7	117.08 (13)	N2—C3—H3A	118.1
N1—C1—C2	121.87 (15)	C2—C3—H3A	118.1
N1—C1—C4	121.19 (16)	N3—C6—C7	122.20 (16)
C2—C1—C4	116.93 (14)	N3—C6—H6	118.9
C6—C7—C10	119.49 (15)	C7—C6—H6	118.9
C6—C7—H7	120.3	N2—C5—C4	124.33 (17)
C10—C7—H7	120.3	N2—C5—H5	117.8
C8—C9—C10	119.61 (15)	C4—C5—H5	117.8
C8—C9—H9	120.2		
			
N4—C10—C7—C6	−178.25 (15)	N1—C1—C4—C5	179.89 (15)
C9—C10—C7—C6	1.2 (2)	C2—C1—C4—C5	−0.6 (2)
N4—C10—C9—C8	178.74 (14)	C5—N2—C3—C2	−0.5 (3)
C7—C10—C9—C8	−0.7 (2)	C1—C2—C3—N2	−0.5 (3)
C6—N3—C8—C9	0.6 (3)	C8—N3—C6—C7	−0.1 (3)
C10—C9—C8—N3	−0.2 (2)	C10—C7—C6—N3	−0.8 (3)
N1—C1—C2—C3	−179.40 (15)	C3—N2—C5—C4	1.1 (3)
C4—C1—C2—C3	1.1 (2)	C1—C4—C5—N2	−0.5 (3)

### Hydrogen-bond geometry (Å, º)

**Table:**

*D*—H···*A*	*D*—H	H···*A*	*D*···*A*	*D*—H···*A*
N4—H4*A*···S^i^	0.86	2.58	3.4222 (17)	165
N4—H4*B*···N5	0.86	2.10	2.952 (2)	168
N3—H3···N2	0.86	1.83	2.688 (2)	172
N1—H1*A*···S^ii^	0.86	2.62	3.4498 (18)	162
N1—H1*B*···N5^iii^	0.86	2.23	3.083 (3)	172

Symmetry codes: (i) −*x*+3/2, *y*−1/2, −*z*+3/2; (ii) −*x*+1/2, *y*−1/2, −*z*+1/2; (iii) *x*, *y*, *z*−1.
